# Comparison of skin carotenoid content and dietary intake between cognitively normal older adults and older adults with Alzheimer’s Disease: an updated matched‐pair analysis

**DOI:** 10.1002/alz.092983

**Published:** 2025-01-03

**Authors:** Jessica E Keller, Debra K Sullivan, Matthew K Taylor, Jeffrey M Burns, Russell H Swerdlow

**Affiliations:** ^1^ KU Alzheimer’s Disease Research Center, Fairway, KS USA; ^2^ University of Kansas Medical Center, Kansas City, KS USA; ^3^ University of Kansas Alzheimer’s Disease Research Center, Fairway, KS USA

## Abstract

**Background:**

Alzheimer’s Disease (AD) is a systemic metabolic disease with a variable number and type of clinical symptoms mostly impacting the brain. Skin carotenoid content (SCC) is an objective measure of carotenoid‐containing fruit and vegetable intake that has been validated in diverse populations. Our previous findings suggest SCC scores differ between older adults with and without AD regardless of dietary intake of carotenoids. Therefore, our objective was to investigate this correlation across a larger sample of matched pairs.

**Method:**

Baseline data from two dietary interventions (NCT03860792, NCT03841539) were analyzed. Pairs are exact matches for sex, race/ethnicity, and education and are matched within three years of age and 5 kg/m^2^ body mass index. Each pair (n = 26 pairs) includes one cognitively normal (MMSE > 25), community dwelling older adult (‘CN’) and one community dwelling older adult with AD (‘AD’). Baseline self‐reported dietary intake data (3‐day food records) were collected, reviewed, and entered into the Nutrition Data System for Research (NDSR 2019) database by a Registered Dietitian. SCC scores were collected through pressure‐mediated reflection spectroscopy via VeggieMeter® (Longevity Link Corporation, Holladay, UT). Paired samples t‐tests were used to compare differences in SCC scores and dietary intake (IBM SPSS, version 29). Significance was set at p = 0.05.

**Result:**

SCC scores were different between matched pairs (SCC scores: AD 194.62, CN 281.50, p = 0.002). There was no difference in dietary intake between AD and CN pairs for daily energy (1768 vs 1824kcal/day; p = 0.75), fat (75.6 vs 78.7g/day; p = 0.71), carbohydrate (196.7 vs 190.0g/day; p = 0.73), or protein (74.3 vs 76.3g/day; p = 0.77), respectively. Both groups had similar food group intake (p‐value range 0.18‐0.84), including all fruit and vegetable groups except CN had higher intake of non‐citrus fruit (p = 0.03; +0.59 servings/day). Individual carotenoid intake was not different between groups (total vitamin A, p = 0.31; beta‐carotene, p = 0.31; alpha‐carotene, p = 0.41; beta‐cryptoxanthin, p = 0.54; lutein+zeaxanthin, p = 0.32; lycopene, p = 0.42).

**Conclusion:**

This updated analysis suggests that individuals with AD have lower skin carotenoid content compared to matched cognitively unimpaired individuals. These differences are not explained by differences in diet suggesting that AD may be associated with disease‐associated biochemical features that are metabolism‐related and not limited to the brain.

